# A Digital Anatomical Atlas of the Human Cerebellum at Subfolial Resolution

**DOI:** 10.1002/hbm.70497

**Published:** 2026-03-11

**Authors:** John G. Samuelsson, Jeremy D. Schmahmann, Martin I. Sereno, Bruce Rosen, Matti S. Hämäläinen

**Affiliations:** ^1^ Mass General BrighamMassachusetts Institute of TechnologyHarvard Medical SchoolHarvard‐MIT Division of Health Sciences and Technology Cambridge Massachusetts USA; ^2^ Athinoula A. Martinos Center for Biomedical Imaging Charlestown Massachusetts USA; ^3^ Ataxia Center, Cognitive Behavioral Neurology Unit, Laboratory for Neuroanatomy and Cerebellar Neurobiology, Department of Neurology Mass General Brigham and Harvard Medical School Boston Massachusetts USA; ^4^ Psychology Department, San Diego State University and Cognitive Science Department University of California San Diego San Diego California USA; ^5^ Department of Neuroscience and Biomedical Engineering, School of Science Aalto University Espoo Finland

## Abstract

Interest in the cerebellum has surged with the emerging consensus that it supports diverse functions that are topographically arranged across the cerebellar cortex. Further refinement of these in vivo structure–function relationships is limited by the resolution of existing atlases. Here we present a digital atlas derived from a recent reconstruction of the human cerebellar cortical surface with a mean inter‐vertex spacing of 0.16 mm, sufficient to accurately trace the contours of the subfolia, while being consistent with the Schmahmann et al. atlas at the lobular level. We also present ARCUS, a diffeomorphic atlas‐to‐subject registration approach that yields an atlas‐derived, lobule‐labeled cerebellar cortical sheet with macroscale folding geometry in individual subjects from standard‐resolution MRI. Publicly released, this atlas offers an anatomical ground‐truth reference in both volumetric and surface representations at unprecedented granularity, enabling novel and more precise analyses and visualizations of cerebellar data.

## Graphical abstract

We present a digital atlas of the human cerebellum at subfolial resolution with both volumetric and surface representations, offering an anatomical ground‐truth reference at unprecedented granularity.

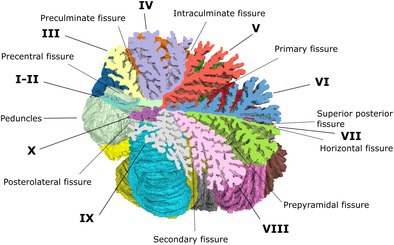

## 1 Introduction

The last few decades have seen a paradigm shift in cerebellar neuroscience and neurology. While the cerebellum has historically been viewed mainly as a motor control center, neuroimaging and clinical case studies have established the cerebellum as a structure containing critical nodes in the distributed neural networks that govern sensorimotor, autonomic, cognitive, and emotional processing with an intricate “fractured” topography (Albus 1971; Andersen et al. 1992; Andreasen and Pierson 2008; Guell, Gabrieli, and Schmahmann 2018; Herculano‐Houzel 2009; Marr 1969; Middleton and Strick 2001; Raymond and Medina 2018; Schmahmann 1991; Schmahmann et al. 2019; Schmahmann and Sherman 1998; Sereno et al. 2020; Shambes et al. 1978; Stoodley and Schmahmann 2009).

Along with our advancing understanding of the wide range of roles the cerebellum plays in health and disease, having access to adequate tools with which to examine cerebellar neurophysiology has become increasingly important. An atlas that can be used to specify and associate anatomical regions of the cerebellum with certainty, enabling data integration across subjects, is one such critical tool. Substantial progress has been made over the past 25 years in the development of cerebellar atlases designed for this purpose. For almost 40 years the definitive atlas of the human cerebellum was that of Angevine et al. (1961). The development of the Talairach atlas (Talairach and Tournoux 1988) to map the human brain in 3D coordinate space using magnetic resonance imaging led to several cerebral cortex parcellations; but the cerebellum initially attracted little interest in this respect.

The branch point in atlasing of the cerebellum for the contemporary era commenced with the publication by Schmahmann et al. (1999) followed up by the more comprehensive MRI Atlas of the Human Cerebellum (Schmahmann et al. 2000). This atlas placed the cerebellum in the Montreal Neurological Institute (MNI) coordinate space, identified the cerebellar lobes, lobules and fissures, and clarified and simplified the nomenclature that had vexed the field for 200 years. The Schmahmann Atlas made it possible to view activations on task‐based MRI (Keren‐Happuch et al. 2014; Stoodley and Schmahmann 2009) and lesion symptom mapping (Stoodley et al. 2016) in the cerebellum with anatomical detail not previously available.

The Schmahmann Atlas also enabled development of the SUIT atlas (Diedrichsen 2006) and subsequent, progressively more sophisticated elaborations of probabilistic and multimodal atlases as well as automated volumetric segmentation algorithms based on advances in computer vision and machine learning, which are now widely used in task‐based, resting state functional connectivity, tractography and morphometric MRI studies of the human cerebellum (Buckner et al. 2011; Carass et al. 2018; Diedrichsen et al. 2009; Guell et al. 2019; Guell, Schmahmann, et al. 2018; Han et al. 2020; Hernandez‐Castillo 2023; Jenkinson et al. 2012; King et al. 2019; Lyu et al. 2024; Makris et al. 2003; Nettekoven et al. 2024; Ren et al. 2019; Romero et al. 2017). Zheng et al. (2023) presented an extremely high‐resolution surface reconstruction of the cerebellar cortex where the lobules were labeled but did not present volumetric representations of the lobules nor were the data from an intact specimen as they were based on the 7404 histological sections from the BigBrain project (Amunts et al. 2013).

As useful and transformative as these atlases have been, there is no available digital cerebellar atlas to the authors' knowledge with resolution fine enough to capture subfolial‐level structures that encompass both volumetric and cortical representations. The availability of such an atlas would be extremely valuable for a wide range of purposes. Some applications include using the cortical surface to constrain the inverse problem in M/EEG source estimation or fMRI analysis. The surface can also be used to visualize neuroimaging data in inflated and flat representations, and the user can interact with these manifolds using rendering software.

**FIGURE 1:**
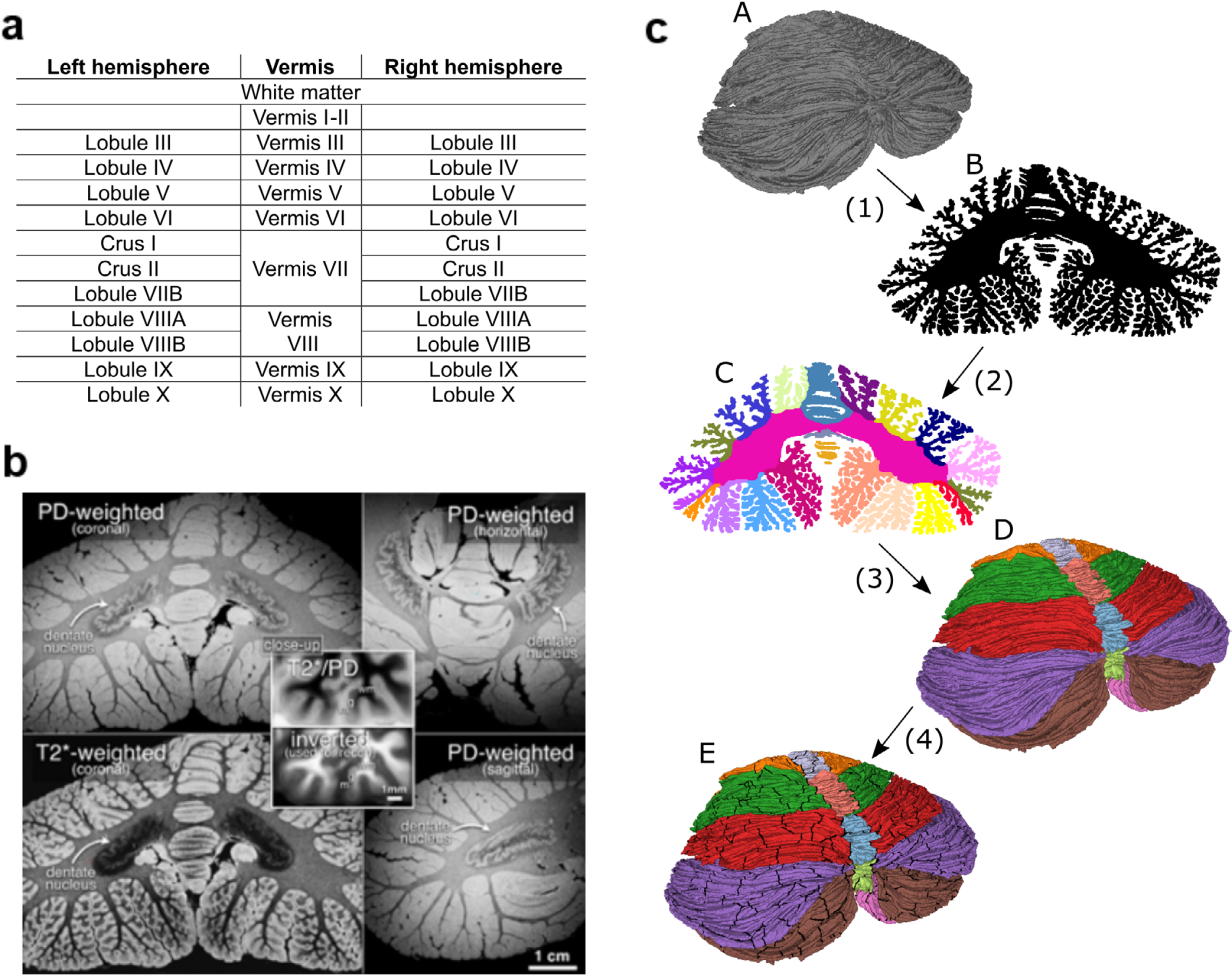
Methodological overview. (a) Regions of our cerebellar atlas. Note that the atlas only involves the cerebellar cortex; cerebellar nuclei are not included. (b) MRI images of the post‐mortem human cerebellum (9.4T, 0.19‐mm‐wide isotropic voxels). The upper and lower left panels show coronal plane proton‐density‐weighted (PD) and T2*‐weighted coronal slices from the two data sets that were combined to reconstruct the surface. The crinkled sheet of the deep cerebellar dentate nucleus is clearest in the proton density‐weighted image (upper left), also visible in the right panels (horizontal and sagittal planes). The central overlay shows extreme closeups of the combined image (T2* divided by PD, then inverted), where thin cerebellar white matter strands (wm) can be distinguished from the deeper granule cell layer (g) and the more superficial molecular layer (m) of the cerebellar cortex. (c) Conceptual schematic outlining the main methodological steps in creating the cerebellar atlas and surface parcellation. (1) Starting from the original surface (A), a virtual volume (B) is created by turning the vertices into voxels and filling the interior of the surface using Trimesh (Dawson‐Haggerty 2019). (2) Anatomical regions in the volume are manually annotated using Freeview software and processed with correction and smoothing filters (C). (3) The volumetric atlas (C) is turned into a surface atlas (D) by assigning each vertex in the surface tessellation to the voxel label in which the vertex resides. The surface atlas is processed with a surface smoothing filter and the consistency between the surface and the volumetric atlas is ensured by labeling each voxel as belonging to the mode of the vertex labels in each voxel. (4) The surface atlas is further subdivided into 688 non‐overlapping patches, resulting in a surface parcellation (E).

To address this need in cerebellar atlasing, we set out to develop a digital atlas of the cerebellum to the resolution of individual cerebellar folia and subfolia using the Sereno et al. (2020) surface as a starting point, and incorporating both volumetric and cortical representations. To make the atlas directly applicable in neuroscience and clinical research, we also present a nonlinear diffeomorphic atlas‐to‐subject registration framework that yields a subject‐space, atlas‐derived cerebellar cortical sheet with lobule‐resolved labels and macroscale folding geometry, providing an approximate (template‐fitted) representation of the cerebellar cortex in individuals. We call this method ARCUS; Automatic Reconstruction of Cerebellar cortex from standard MRI USing diffeomorphic registration of a high‐resolution template. This is done by first segmenting the lobules of individual cerebella, then registering the volumetric atlas to the subject space, then applying the transformation fields from these registrations to the vertices in the surface mesh. ARCUS is explained in further detail in the Methods section. Figure 1b illustrates several MRI slices from the post‐mortem cerebellum scans from which that surface reconstruction was made, demonstrating the clear visualization of subfolial detail.

## 2 Results

### 2.1 Atlas

**FIGURE 2:**
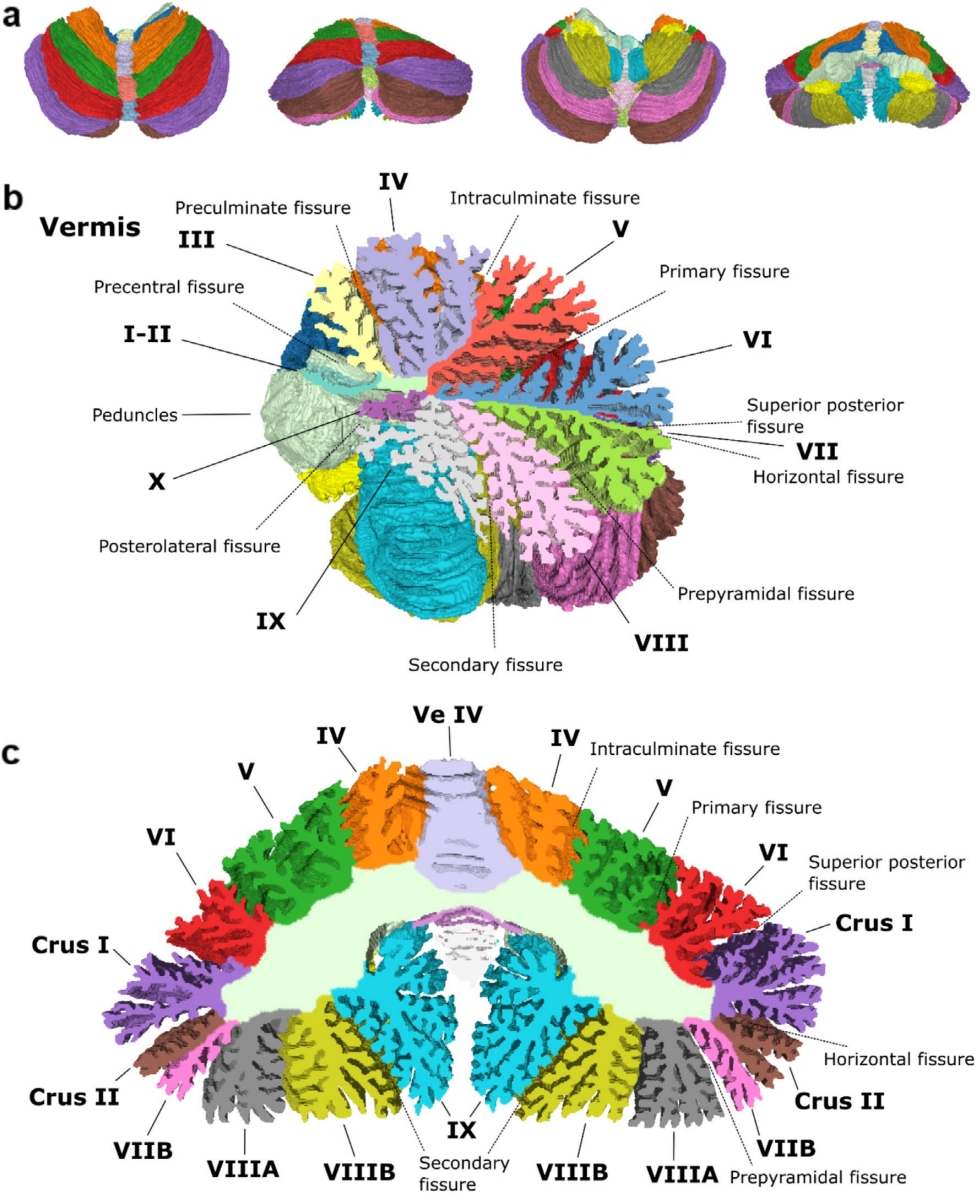
Cerebellum atlas (a) Cerebellum surface atlas with lobules color coded (see c for labels). (b) Sagittal cross‐section at the midline, displaying the volumetric atlas together with the surface atlas of the right half. (c) Coronal cross‐section showing the volumetric atlas together with the anterior half of the cerebellar surface atlas.

The atlas has both a volumetric and a cortical surface representation. The surface representation is further parcellated into 688 non‐overlapping small patches, as detailed in Methods. The surface atlas is shown in Figure 2 from different angles along with a sagittal cross‐section around the midline and a coronal cross‐section where the cuts have been filled with the corresponding volumetric atlas data. The surface atlas is mostly delineated by the cerebellar cortex. A closed surface is made by including the cut surfaces of the superior, middle and inferior cerebellar peduncles and a small stretch of their exposed stalks in the fourth ventricle, annotated here as white matter tracts. There were also smaller patches of exposed white matter at various locations in the specimen but they were not labeled as such as to not have a discontinuous atlas. The nomenclature is derived from the Schmahmann Atlas, and the anatomical regions are listed in Figure 1a. The cerebellar fissures are also annotated in the Figure with two exceptions; the ansoparamedian fissure separating Crus II and Lobule VIIB, and the intrapyramidal fissure separating Lobules VIIIA and VIIIB. These fissures were not annotated because they were inconsistent between the left and right hemispheres; the fissures separating these lobules “jumped” between folia around the midline. While the horizontal fissure separating Crus I and Crus II is consistent between the hemispheres and therefore marked, it is diminutive at the midline and barely detectable.

### 2.2 Alternative Representations

**FIGURE 3:**
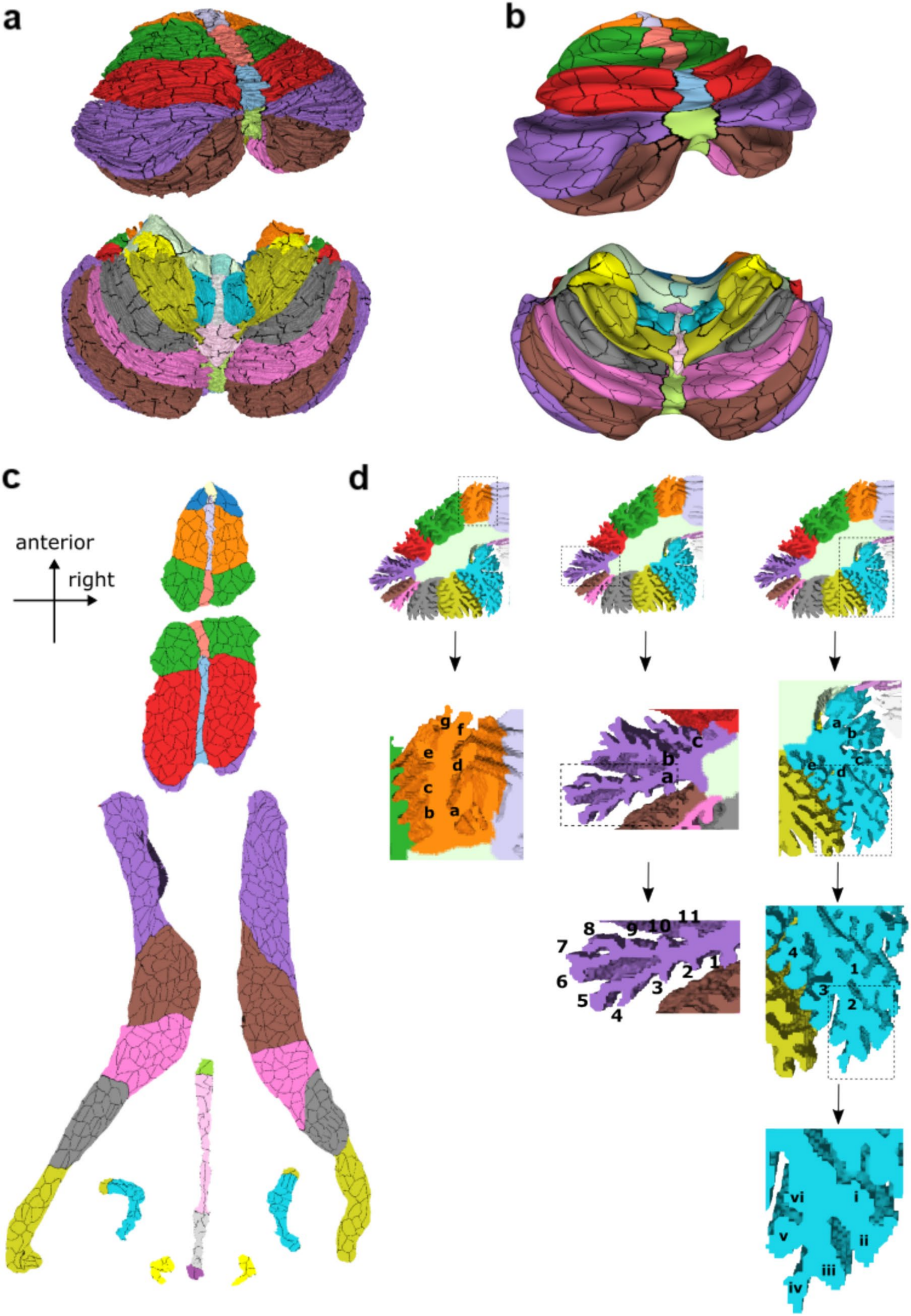
Finer surface parcellation and alternative representations. Surface atlas with patch parcellation borders marked in black on different representations: Normal (a), inflated (b) and flat (c). Note that the scale in (a, b, and c) differs; (a) is largest, (b) is next, and (c) is the smallest. (d) Enlarged cross‐sections in the coronal plane. Left column shows lobule IV, middle column Crus I and the right column lobule IX. The folial branching steps have been labeled in the rows; lowercase letters denote the first branching (second row), numbers the second branching (third row) and lowercase Roman numerals the third branching (fourth row).

Figure 3a–c shows the surface atlas in inflated and flat representations along with the parcellation where the boundaries between the small patches are marked in black. Figure 3d shows enlarged images of lobule IV, Crus I, and lobule IX. The figure displays the elaborate branching of the cerebellar lobules. While the number of folia and subfolia varies across lobules and cross‐sections, the branching pattern differs as well. Lobule IV has one main folium in the cross‐section shown in Figure 3d which branches into the subfolia, whereas Crus I has three main folia which branch into smaller branches before branching again into the subfolia. This recursion is even more elaborate in lobule IX, where even more branching steps are evident, three of which are shown in the figure, denoted with different numerals.

### 2.3 Surface Areas and Folial Inconsistencies

**FIGURE 4:**
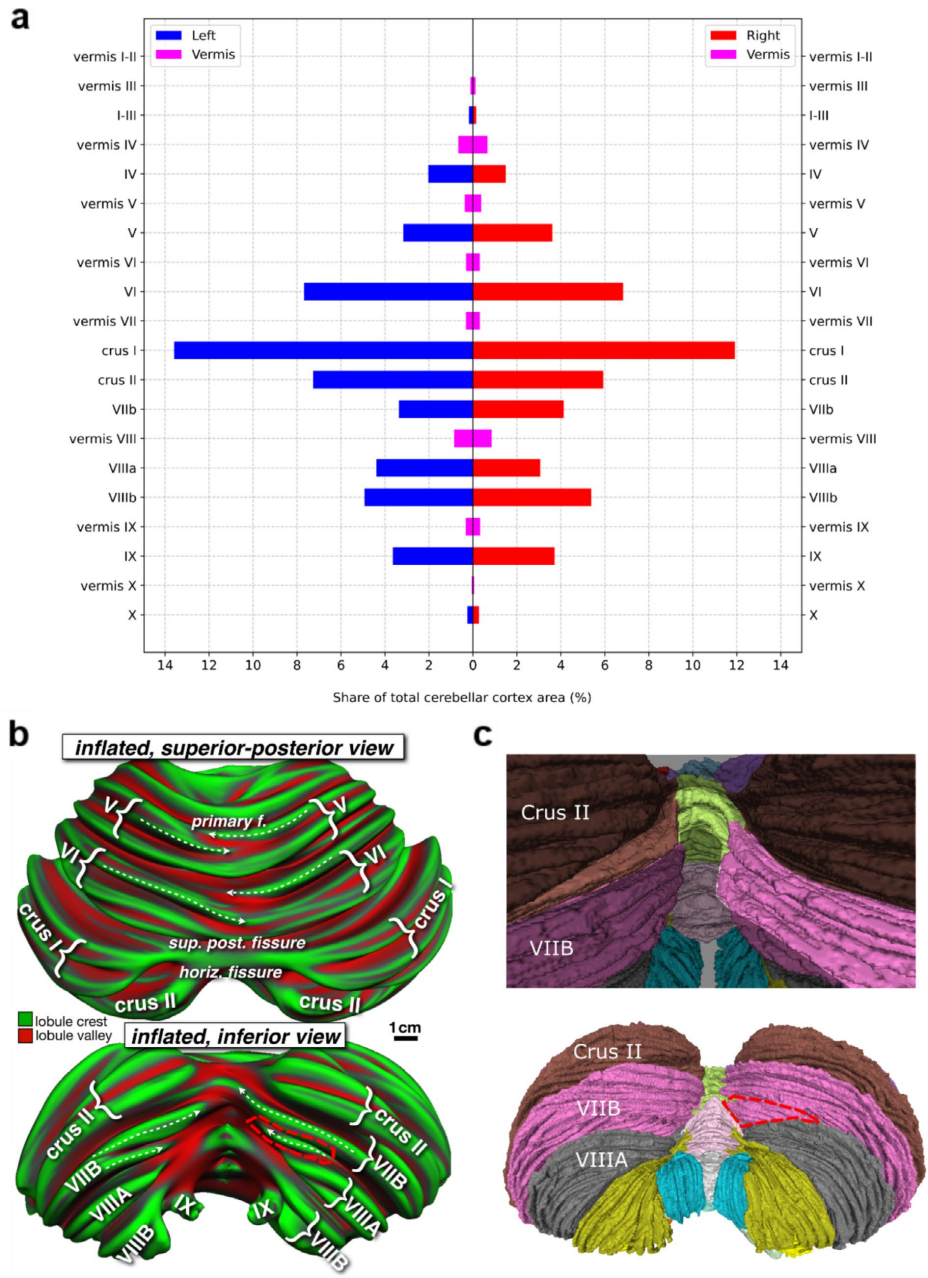
Surface areas and subfolial lobule switching. (a) Relative surface area of cerebellar lobules as a percentage of total cerebellar cortical surface area; the sum of left, right and vermis is 100%. The vermis area has not been divided into left and right; it is visualized leftwards and rightwards only for illustrative purposes. (b) Lobular geometry at the midline in superior‐posterior view (top panel) and inferior view (bottom panel). The cerebellar surface has been inflated to unfold the numerous folia and then colored by FreeSurfer average convexity (“sulc”), which mostly ignores the tiny folds of individual folia and fissures and instead marks major lobule crests and valleys. (c) Posterior‐inferior view of the cerebellar atlas. The upper image shows the boundaries of crus II and lobule VIIB in relation to the midline. The lower image shows what would remain of lobule VIIB (outlined in red dashes) if we had assiduously followed the ansoparamedian fissure across the midline from left to right without recognizing that small midline fissures may not be continuous across the hemispheres and thereby connect the “wrong” lobules. This highlights one example of the substantial lateral asymmetry that could arise by adhering strictly to the paradigm of letting fissures demarcate all lobule boundaries.

The relative surface areas of the cerebellar lobules as a percentage of the total cerebellar cortical area are outlined in Figure 4a. Crus I is the largest lobule, representing almost 14% (left) and 12% (right) of the cerebellar cortical surface area. The smallest lobule is Vermis I‐II, representing only 0.01% of the surface area. The anterior, posterior, and flocculonodular lobes occupy 11.8%, 87.6%, and 0.6%, respectively.

Figure 4b shows an inflated view of the convexity of the cerebellar cortex, illustrating the positioning and size of the folia. The dashed arrows in the top panel show that left and right folia are not always continuous across the midline but rather appear interleaved and sometimes continuous with the “wrong” folia/lobule in the other hemisphere. In the lower panel, the dashed arrows show that folia crests are tilted with respect to the main axis of the lobule and may disappear well before reaching the midline. The ansoparamedian and intrapyramidal fissures were inconsistent between the left and the right hemispheres. To illustrate this point, the ansoparamedian fissure along with the folium that “switches” labels around the midline is highlighted in Figure 4c.

### 2.4 Arcus

The unweighted median DICE score (of the medians across subjects) between the volumetric lobular segmentations and the manually annotated lobules was 0.791 and the unweighted mean score (of the medians across subjects) was 0.765. The lowest median score was 0.553 for right lobule V and the highest was 0.932 for left Crus I. Distributions of subject‐level DICE scores across three structural hierarchies are shown in Figure S1.

**FIGURE 5:**
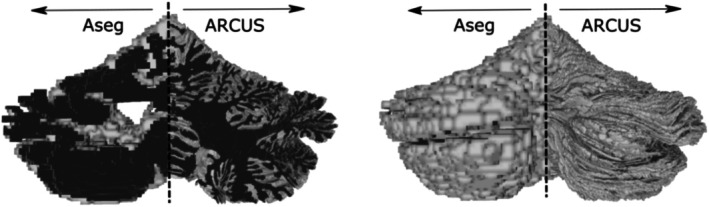
Comparison of ASEG and ARCUS. A representative example of a subject reconstruction using ARCUS compared to the reconstruction produced when tessellating ASEG's cerebellar cortex segmentation.

Hausdorff distances between the surface reconstructions and the annotated volumes, evaluated across the same three structural hierarchies, are shown in Figure S2. At the lobular/vermis level, Hausdorff distances ranged from 2.8 to 20.1 mm (IQR 5.4–8.7 mm), with a mean of 7.2 mm, median of 7.1 mm, and standard deviation of 2.4 mm. Figure 5 shows a representative reconstruction from standard‐resolution (1 mm isotropic) in vivo MRI, alongside a surface obtained by tessellating the FreeSurfer ASEG cerebellar cortex segmentation (coronal view) and the corresponding outer surface.

## 3 Discussion

Human cerebellar atlases have been pivotal in the transformative studies that have led to deeper understanding of the cerebellum and its multiplicity of functions in the nervous system (Schmahmann et al. 2019). The contemporary topographical divisions of the cerebellar cortex that were employed in this study (Figure 1a) date back to the early descriptions of the cerebellum (Bolk 1906) and were reaffirmed with extensive comparative morphological studies (Larsell 1958; Larsell and Jansen 1972) and detailed anatomical and physiological investigations in experimental models (Chambers and Sprague 1955; Jansen and Brodal 1954; Oscarsson 1965; Voogd 2003). The range and complexity of cerebellar functional incorporation into the distributed neural circuits subserving neurological function and the precision of the topographic arrangement of cerebellar connections and functional topography have become increasingly apparent. Consequently, the need for a digital cerebellar atlas that enables both surface and volumetric representations with resolution high enough to resolve the anatomy of the cerebellar cortex at the level of the individual folia and subfolia has become more pressing. Here we aimed to address this need by utilizing the recent high‐resolution surface reconstruction of the cerebellar cortex presented in Sereno et al. (2020) to create an atlas and developing a method for morphing this atlas to individual subject space.

Our digital atlas provides a means for the user to interact with the atlas in 3D rendering software. It is possible to rotate, zoom, slide, or choose to see specific regions of the atlas, providing an intuitive way of understanding cerebellar anatomy at a highly granular level. Other digital volumetric atlases such as SUIT (Diedrichsen 2006) also allow for such interactions using graphics rendering software, but the unique advantage of the atlas presented here lies in the highly detailed surface representation together with the volumetric representation. This combined surface‐volumetric atlas enabled ARCUS, which diffeomorphically warps the atlas into individual subject space to yield an atlas‐derived, high‐resolution, lobule‐labeled cerebellar cortical sheet—an approximate, template‐fitted representation of each subject's cerebellar cortex. ARCUS is highly relevant within neuroimaging research as surface reconstructions are essential in studies that use a cortical manifold, e.g., in M/EEG source estimation and forward modeling (Samuelsson, Sundaram, et al. 2020). ARCUS has already shown success in early demonstrations in our research center, e.g., (Samuelsson 2021) integrated it with MNE‐python, providing a software toolkit for cerebellar M/EEG source modeling. These techniques have in turn been used in various other projects, e.g., to study cerebro‐cerebellar functional connectivity changes in epilepsy and autism as well as been employed in cerebellar TMS modeling (Alho et al. 2023; Samuelsson, Rosen, and Hamalainen 2020). ARCUS also enables intuitive visualizations of topographic data of the human cerebellar cortex in vivo since it has inflated, flat and spherical representations.

Importantly, ARCUS is not intended to achieve the pointwise surface accuracy attainable for the *cerebral* cortex when reconstructing pial/white‐matter boundaries from anatomical MRI (e.g., FreeSurfer's boundary‐based surface reconstruction (Fischl 2012)). An analogous boundary‐driven approach is currently impractical for the tightly folded cerebellar cortex at contemporary in vivo MRI resolutions; Sereno et al. (2020) suggested that properly resolving folial contours requires isotropic voxel sizes below 0.2 mm. Instead, ARCUS diffeomorphically fits a high‐resolution atlas to subject space using lobular segmentations and local image contrast, yielding an approximate, template‐fitted cerebellar cortical sheet that preserves lobule‐specific folding geometry. This provides a more anatomically informative surface than direct tessellation of a cerebellar gray‐matter segmentation, which typically approximates the outer envelope and fails to capture sulci and deep fissures (Figure 5).

While ARCUS showed promising performance on the manually annotated volumetric dataset (median DICE 0.791; median Hausdorff distance 7.1 mm), further validation across scanners, protocols and populations is warranted. Since subject‐specific ground‐truth cerebellar surfaces that capture the subfolia from in vivo MRI are not currently available, Hausdorff distances were computed between a voxelized representation of the reconstructed cortical sheet and the filled manual lobular volumes; consequently, these values reflect a surface‐volume discrepancy and may be inflated relative to a boundary‐only surface‐surface Hausdorff comparison. Still, the resulting surfaces may be insufficient for applications with very high spatial‐accuracy demands (e.g., fine‐grained morphometry or high‐field fMRI targeting folial‐scale features). However, for applications whose effective spatial resolution is coarser, such as distributed M/EEG source modeling (≈5–20 mm; Samuelsson et al. 2021), ARCUS should provide an adequate and substantially improved cerebellar geometric substrate relative to envelope‐like alternatives.

In annotating the atlas, we encountered and revisited two major anatomical issues discussed explicitly in Schmahmann et al. (1999) and Schmahmann et al. (2000). First, the authors discussed what they called the problem of the vermis, noting that there is indeed no true “vermis” in the anterior lobe. They noted that the application of this term to the paramedian sectors of the anterior lobe term is an extension of the Latin term (meaning worm) used by Malacarne (1776) to denote the structure visible in the posterior and inferior aspect of the cerebellum. While the vermis as an anatomically defined entity is morphologically distinct only in lobules VII‐X, vermis as a midline concept without a clear anatomically defined lateral boundary in lobule VI and the anterior lobe is well established in the literature. In this atlas, we outlined a vermis in lobules I–VII based on subtle changes in folia width, folia discontinuities, and shallow paramedian creases visible in the folded cortex.

The second issue that we revisited was the definition of cerebellar lobules according to the arrangement of the cerebellar fissures. The lobules of the cerebellum are delineated by deep fissures between separate groups of folia that cross the midline from one hemisphere to the other. This approach builds on the seminal observations by Bolk (1906) and Larsell (1958) and was crystallized in Schmahmann et al. (1999) and Schmahmann et al. (2000). In the present study we observed that the ansoparamedian fissure separating crus II from lobule VIIB violated this paradigm by crossing lobule boundaries near the midline. The same was true for the intrapyramidal fissure separating lobules VIIIA and VIIIB. It was already noted in Sereno et al. (2020) that the axes of individual folia are often tilted with respect to the overall axis of a lobule; Sereno et al. (2020), Figure 4., shows folia near the base of several lobules progressing toward lobule crests as one moves medially. An analogous observation was also made by Zheng et al. (2023). Figure 4 in the present manuscript expands on that theme, showing the complex geometry of sub‐lobule crests at the midline, where they often appear to be interleaved rather than continuous across the midline. Subfolia switching labels is not constrained to the midline; indeed, several subfolia switch labels and “flow into” another lobule as one moves laterally in a sagittal cross‐section view.

The alternative of strictly adhering to the paradigm of letting only fissures delineate lobule boundaries, and therefore annotating a folium as belonging only to lobule VIIB or crus II (or VIIIA or VIIIB) without a change of label in the vermis, yielded substantial asymmetry in the lobule sizes between the left and right hemispheres (Figure 4). The synopsis of this observation is that the ansoparamedian fissure that separates crus II from lobule VIIB can be difficult to ascertain with certainty. An “ansoparamedian lobule” that includes both crus II and lobule VIIB was discussed by Larsell and von Berthelsdorf (1941), but we do not recommend this designation, preferring the agnostic approach of acknowledging that if there is indeed asymmetry across the hemispheres of crus II and lobule VIIB, this invites future exploration of the potential functional and clinical relevance of the observation.

The flocculi (lobules X) in the present atlas are not connected to the nodule (vermis X) which goes contrary to the idea of a “flocculonodular lobe”. This is because there is no morphological feature that connects them; the posterolateral fissure that divides lobules IX and X in the vermis disappears as one moves laterally.

An interesting observation is that the folial branching pattern seems to be different across different lobules. While some lobules, e.g., lobule IV in Figure 3, have a relatively straightforward branching pattern with one folium directly branching out into subfolia, some lobules like lobule IX in Figure 3 have a much more elaborate branching pattern with several branching recursions before branching into the subfolia. The general trend seems to be that the posterior cerebellum has a higher rate of these branching recursions than the anterior lobe. This is part of the reason why the area of the cerebellar cortex of the posterior lobe is so much larger than the area of the anterior lobe; the posterior lobe occupies as much as 88% of the total cerebellar surface area in our specimen.

The presented atlas limits its focus to the cerebellar cortex. The cerebellar nuclei and the fiber tracts in the cerebellar white matter, the *corpus medullare*, have not been annotated. While these could potentially be added in future versions of the atlas, the power of the present analysis lies in the unparalleled detail of the morphometry of the reconstructed cerebellar cortex. Note that cerebellar white matter extends fully into the subfolial regions (Figure 1). Accordingly, the structure labeled as the Corpus Medullare (CM) in our volumetric segmentation should not be interpreted as the complete extent of white matter. Instead, the CM–lobule boundary reflects a computational rather than a strictly anatomical distinction. In cerebellar MRI segmentation, this boundary is usually defined around the point where the lobular branches emerge from the central white matter. Maintaining this convention is essential for reliably deforming the atlas to individual brains, as it ensures consistency between the volumetric atlas and the individual volumetric segmentations.

As in Schmahmann et al. (1999) and Schmahmann et al. (2000), we studied a single brain. Whereas this approach inherently includes idiosyncrasies of one individual's cerebellar morphometry, its power lies in defining discrete anatomical features that could be functionally and clinically relevant but potentially overlooked when using the averaging and smoothing algorithms necessary for the analysis of large numbers of brains. The goal of this study was to create a ground truth atlas at a previously unattained resolution for use as a template and reference in future studies.

The atlas is based on the surface reconstruction that was presented in Sereno et al. (2020), in which the geometry of the human cerebellar cortex was reconstructed to the level of all individual folia and subfolia for the first time. Thus, the limitations described in Sereno et al. (2020) also apply to the present atlas. This includes potential drawbacks of reconstructing a specimen ex vivo, e.g., tissue swelling and the potential morphological differences introduced when the tissue is removed from the hydrostatic pressure of the cerebrospinal fluid in the intact skull followed by fixation‐induced shrinkage.

The finer cortical parcellation presented in this work that subdivides the 32 regions of the atlas into 688 smaller patches was not based on anatomical or functional criteria as was done, e.g., in Makris et al. (2003) and Makris et al. (2005), but rather on a semi‐stochastic algorithm to create a set of non‐overlapping patches of roughly equal size. The finer parcellation should therefore be viewed mainly as a tool to more precisely describe locations of functional or anatomical data gathered by other means than would be possible with the original 32 region atlas. Furthermore, the average patch size was chosen so that they would be approximately equivalent to the size that would generate a detectable MEG/EEG signal when activated synchronously, making them useful in MEG/EEG modeling, such as that proposed in Samuelsson et al. (2021).

## 4 Conclusions

We developed a new digital anatomical atlas of the human cerebellum with surface and volumetric representations, based on the first reconstruction resolving individual folia and subfolia. We also introduced ARCUS, a method that diffeomorphically maps the atlas to individual brains to yield an atlas‐derived, lobule‐labeled cerebellar cortical sheet that preserves lobule‐scale folding geometry from standard‐resolution in vivo MRI. As with advanced atlases of the cerebral cortex, this atlas has unfolded and flattened planar representations. With its unprecedented granularity, detailed manual labeling, and digital format, it can serve as an expert‐annotated anatomical reference, enabling new ways of analyzing and visualizing anatomical and physiological data in the human cerebellum.

## 5 Materials and Methods

### 5.1 Overview

The anatomical regions that this atlas defines are listed in Figure 1a. The cortical atlas representation with 32 anatomical regions was further subdivided into 688 smaller patches.

### 5.2 Specimen Preparation and Scan Parameters

A cerebellar specimen (62 year old female, death from cardiac arrest, without history of previous trauma or neurological conditions, width 96 mm) was obtained from the Netherlands Brain Bank, which has obtained official ethical approval for its procedures as well as informed consent from donors, which include informed consent to donation (https://www.brainbank.nl/about‐us/ethics/). The brain was removed from the skull within 6 h of death. The cerebellum was removed by cutting the cerebellar peduncles and then fixed by immersion in formalin. No gadolinium was added.

After 4 months in formalin, the cerebellum was placed in a tightly fitted cylindrical acrylic chamber. The chamber was filled with perfluoropolyether (Fomblin), which has no ^1^H protons so it has no signal (and no noise) on standard MRI images. The chamber was then pressurized to one atmosphere above ambient air pressure and refrigerated for a week to cause bubbles to dissolve. The chamber was then brought to room temperature and placed inside a tightly fitting quadrature RF coil (6 cm inside diameter). An RF analyzer was used to hand tune the quadrature coil with the specimen in place for maximum imaging sensitivity. The specimen was then scanned in two separate overnight sessions a week apart on a 9.4T MR scanner (Agilent Technologies, Santa Clara, CA, USA). Data from the second session alone (which had better‐optimized scan parameters) was used for the surface reconstruction. That scan session included short and long echo time (TE) standard 3D gradient echo (FLASH) sequences (short echo time proton‐density‐weighted (PD): flip = 10 deg., TE = 3.7 ms, TR = 15 ms; long echo time effective transverse relaxation time‐weighted (T2*): flip = 20 deg., TE = 18 ms, TR = 30 ms; both scan sets used matrix: 512 × 340 × 340; isotropic 0.19 × 0.19 × 0.19 mm voxels; NEX = 10; total time: 12 h). Further details on surface reconstruction and head‐removal‐dependent and fixation‐dependent shrinkage correction can be found in the Supplementary text in Sereno et al. (2020).

That Supplementary text also contains an extensive discussion of the current sample in relation to an average human cerebellum. Briefly, the volume of the convex hull of the reconstruction was compared to average volumes from the literature, including the fsaverage cerebellum volume. The gray matter volume estimate was also compared to estimates from the literature. Overall, these two methods suggest that the volume of the current cerebellum specimen is not at all untypical. Furthermore, the folial features, sulcal features and asymmetries observed in this specimen were also observed by one of the authors (JDS) during preparation of a previous, lower resolution atlas (Schmahmann et al. 1999).

### 5.3 Creation of the Atlas

The atlas was created by a combination of manual annotation and image processing of the cerebellar surface reconstruction presented in Sereno et al. (2020). The spatial resolution of that reconstruction, and thus also the derived atlas presented here, is about 0.16 mm in edge length between neighboring vertices in the surface representation. The volumetric representation of the atlas was calculated from the surface representation maintaining the resolution of the surface. An overview of the process for creating the cerebellar atlas is outlined in Figure 1c, and the detailed account follows.

Starting from the surface tessellation containing 4′573′612 vertices and 9′163′916 triangular faces in Sereno et al. (2020), the surface was down‐sampled and turned into a volume by computation of solid angles in a regular volumetric grid. The down‐sampled volume was manually annotated in Freeview (Fischl 2012) and the volumetric annotations were assigned back to the down‐sampled surface; the atlas was thus not created through registration to a prelabelled volume, but was manually annotated in volumetric space one cross‐section at a time. The down‐sampled surface labels were then scaled to the original high‐resolution surface which were subsequently manually corrected for any inadequacies resulting from the scaling. A virtual volume was then created using Trimesh (Dawson‐Haggerty 2019), preserving the resolution of the original surface, and the labels from the surface were transferred to the volume. The inner virtual volume was filled, and each anatomical region was expanded at the expense of the inner volume in 7 iterations so that the voxels in the folia were labeled according to their cortical annotation. The remaining inner‐volume voxels were annotated as white matter. The labeled volume was then smoothed in 3 iterations where each voxel was assigned the mode of the neighboring voxels. The white matter volume was then expanded in 4 iterations to make sure that the lobule labels did not penetrate too deep into the *corpus medullare*. The voxels that contained a vertex of the surface were then again assigned the labels of the surface to ensure consistency with the surface atlas following the expansion of the white matter. All non‐white‐matter labels were then expanded once again at the expense of the white matter. The vertices in the surface atlas were then assigned the label of the voxel which contained them, the surface was smoothed and the surface atlas was assigned back the volume atlas. Finally, a few manual corrections were made resulting in the final surface and volume atlas.

### 5.4 Parcellation of the Surface Atlas

The surface atlas, containing 32 distinct anatomical regions, was further subdivided into 688 patches of approximately equal size (CV = 42%, Range Ratio = 6.7). This was done by first defining the number of patches in each region to $800\times \frac{\text{area of region}}{\text{total area}}$ for an initial parcellation of 800 patches. The number of patches was chosen so that the area of the patches would be approximately equal to the patches in the Laussanne parcellation at the finest scale (Cammoun et al. 2012); the Laussanne parcellation contains 1000 patches and the cerebellar cortex is roughly 80% of the area of the cerebral cortex (Sereno et al. 2020). Note that the surface patches are roughly two orders of magnitude larger than the small (1–2 mm^2^) “fractured somatotopy” patches mapped by Shambes et al. (1978) and Kassel et al. (1984) with microelectrodes.

A number of starting vertices equaling the number of patches in each anatomical region were randomly chosen. These vertex pointers were then moved away from one another in an iterative fashion. This was done by moving each pointer from its vertex to the neighboring vertex that was furthest away in terms of geodesic distance and that belonged to the same anatomical region, thus spreading the pointers over the cortical manifold. The geodesic distance was approximated as the Euclidean distance in a spherical surface representation of the surface mesh. This was done for each pointer in 100 iterations.

The patches were then created by recursively expanding each patch from the starting vertices by including each neighbor that had not yet been assigned to a patch. This expansion continued until all vertices had been assigned to a patch. To make the patches more circular and avoid narrow strips, the surface division was smoothed using the same technique as described above for the surface atlas; each vertex was labeled as the mode label of all vertices connected to that vertex by three edges or less in three iterations. All patch areas were then calculated and the patches with a surface area larger than twice the mean surface area were split into two. The patches with a surface area less than half of the mean surface area were merged with the neighboring patch with which it shared its longest border. This was done in an iterative fashion. This procedure did not converge for all patches (a maximum of 5 merges plus splits were allowed for each patch) and a final iteration was made where all large patches (surface area > twice the mean) were split once and all small ones (surface area < half of the mean) were subsequently merged, resulting in a final total of 688 patches.

### 5.5 Arcus

A full account of ARCUS can be found in the S1. Briefly, the method has two main steps: segmentation and cortical surface reconstruction by diffeomorphic registration of the atlas.

**FIGURE 6:**
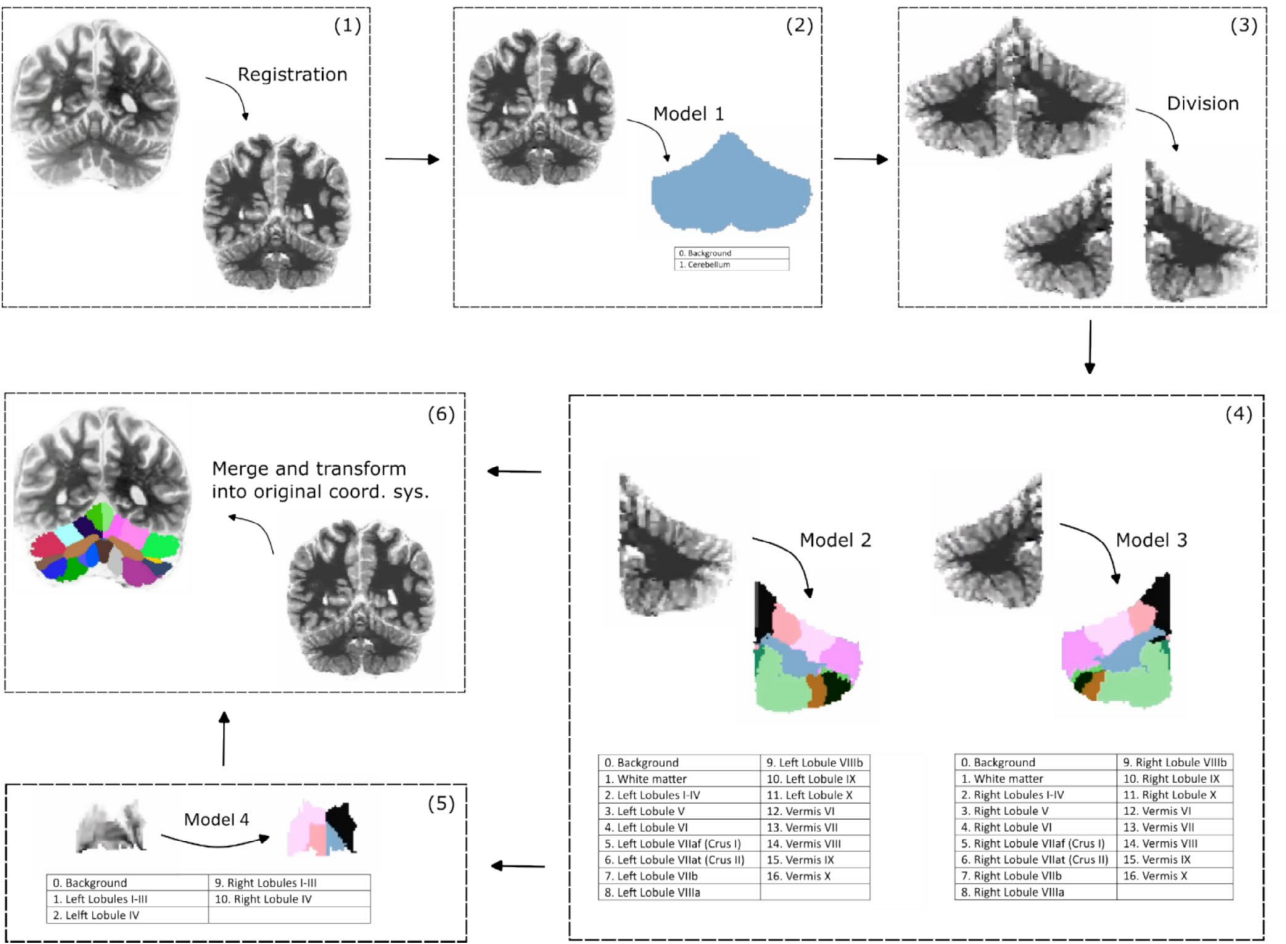
ARCUS segmentation workflow overview. Schematic overview of the segmentation algorithm outlining the sequential steps in the ARCUS segmentation workflow.

In the segmentation step, the lobules of the cerebellum are segmented in a volumetric subject MRI image. This is achieved in a hierarchical procedure that combines several trained U‐net models and heuristic algorithms. An overview of the segmentation step is shown in Figure 6 (Avants et al. 2009; Carass et al. 2018; Collins et al. 1994; Fischl 2012; Isensee et al. 2021; Ronneberger et al. 2015; van der Kouwe et al. 2008).

**FIGURE 7:**
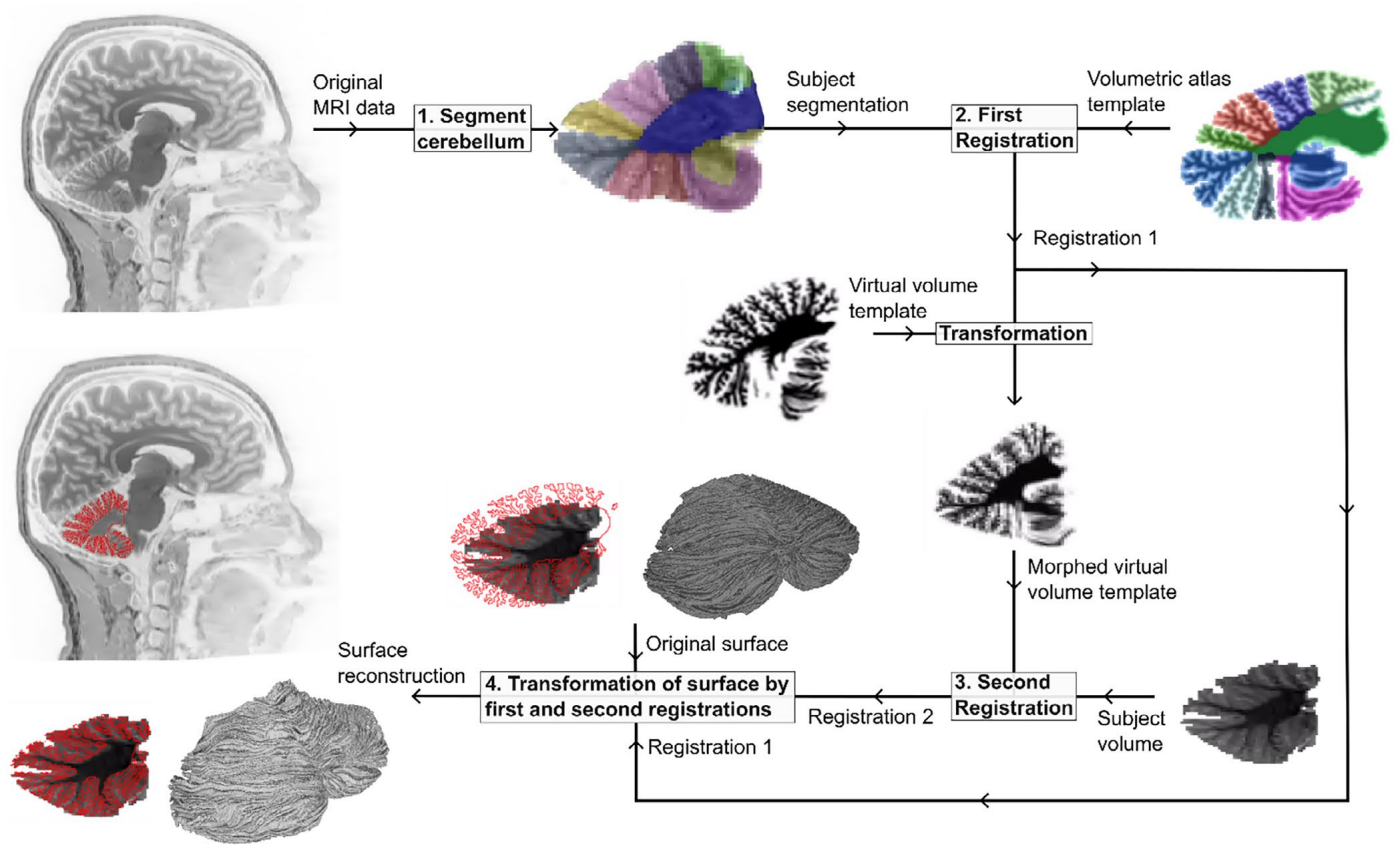
ARCUS reconstruction workflow overview. Conceptual schematic outlining the atlas‐to‐subject mapping. (1) The cerebellum is segmented using the procedure outlined in Figure 6. (2) The volumetric atlas is registered to the subject segmentation. The transformation is saved and applied to the virtual MRI volume of the atlas. (3) The transformed virtual MRI volume of the atlas is registered to the subject cerebellum in a second registration. The transformation is saved. (4) The two transformations resulting from the first and second registrations are applied to the atlas surface, registering the surface to the subject and thus resulting in the cortical sheet in native subject space.

In the reconstruction step, the volumetric atlas is first registered to the subject segmentation. This ensures overall lobule fit. In the second step, the first registration is applied to a virtual MRI image of the volumetric atlas and the registered virtual MRI image is then registered to the masked subject image. This second registration fine‐tunes the fit. The transformation fields of these two registrations are then applied to the vertices in the surface atlas, resulting in a morphed surface atlas mesh that has been fitted to the subject space based on the subject MRI image and thus yielding an approximate reconstruction of the cerebellar cortex in individual brains. The registrations are done using the non‐linear deformation procedure symmetric normalization with cross‐correlation as the optimization metric as implemented in the ANTs software package (Avants et al. 2011). An overview of the reconstruction methodology is shown in Figure 7.

To train the U‐net models and evaluate ARCUS, we used data from Carass et al. (2018) and Diedrichsen et al. (2009), comprising a total of 34 labeled cerebella from standard‐resolution MRI data (1 mm isotropic voxel size). The evaluation scores were calculated in three different hierarchies: (1) coarse; the whole cerebellum, the white matter and the whole vermis, (2) lobe; lobules divided into left and right anterior lobe (lobules I–V), superior posterior lobe (lobules VI, VII), inferior posterior lobe (lobules VIII, IX) and flocculonodular lobe (lobule X), (3) vermis and hemispheric lobules; all 28 divisions in the segmentation excluding the corpus medullare.

## Author Contributions

J.G.S. conceived the study, performed manual labeling and analysis, and wrote the manuscript. J.D.S. guided the study, reviewed labeling, and co‐authored the manuscript. M.I.S. guided the study, performed manual labeling and analysis, provided surface reconstruction data, and co‐authored the manuscript. B.R. guided the study and co‐authored the manuscript. M.S.H. guided the study and co‐authored the manuscript.

## Funding

This research was funded by the NIH Neuroimaging Training Program (NTP) grant 5T32EB001680, NIH/NIBIB P41 Center for Mesoscale Mapping (1P41EB030006), NIH/NINDS grant 5R01NS104585, the NIH BRAIN Initiative F‐32 Fellowship grant 1F32MH127789, NIH Grant R01 MH081990 and UK Royal Society Wolfson Fellowship (M.I.S.). Supported in part by the National Ataxia, Great Oaks, Once Upon A Time, ARSACS and MINDlink Foundations (JDS). The content is solely the responsibility of the authors and does not necessarily represent the official views of the National Institutes of Health.

## Conflicts of Interest

The authors declare no conflicts of interest.

## Supporting information


**Data S1:** Supporting Information.

## Data Availability

All atlas data are available for download at https://osf.io/98p3a/?view_only=933654b10152444992b9e7d8ff9f1112 and the original full resolution volume and surface data are available at https://pages.ucsd.edu/~msereno/cereb. ARCUS can be accessed at https://github.com/johnsam7/ceremegbellum.git. The image processing scripts used in the creation of the atlas are available from the corresponding author upon reasonable request.
