# Supplementary material for: A Digital Anatomical Atlas of the Human Cerebellum at Subfolial Resolution
Source: Hum Brain Mapp. 2026 Mar 11;47(4):e70497. doi: 10.1002/hbm.70497 (PMC12977127; doi:10.1002/hbm.70497)
Supplement: Supplementary file 1 — Data S1: Supporting Information. [file HBM-47-e70497-s001.docx]

# Supplemental Material

## ARCUS Segmentation

### Algorithm

The segmentation method assigns a categorical number representing a cerebellar region to each voxel in reconstructed volumetric subject images (256x256x256 unsigned 8 bit integer) acquired using the multi-echo Magnetization Prepared Rapid Gradient Echo (MEMPRAGE) pulse sequence (van der Kouwe et al., 2008). This method works in a hierarchical fashion; an overview is presented in Figure 6. The segmentation can be summarized in the following six steps:

1. The subject image is registered to the MNI Average Brain, consisting of an average of 305 T1-weighted head MRI scans, linearly transformed to Talairach space (Collins et al., 1994), using ANTS (Avants et al., 2009). The registration is an affine followed by a deformable transformation with cross-correlation as the optimization metric.
2. The cerebellum in the registered image is found using a U-net model and the image is masked (Ronneberger et al., 2015).
3. The masked cerebellum is divided into left and right, and then cropped. This division is done registering the subject’s ASEG (Fischl, 2012) segmentation to the common space and then using the registered left/right cerebellar division as defined by ASEG. The voxels determined to be a part of the cerebellum by the U-net model mask from step 2 but not by ASEG are assigned to left or right cerebellum by iteratively assigning the mode of the neighboring voxels that have already been assigned to left or right cerebellum, thus “spreading” the left/right classification from the assigned to the unassigned voxels.
4. The images of the left and right cerebellum are masked using the right/left division from step 3 and segmented separately using two different U-net models.
5. Lobules I-IV are separated into lobules I-III and lobule IV by first merging left and right lobules I-IV into one image and then segmenting that image into left and right lobules I-III and lobule IV using another U-net model.
6. The segmentations are merged and transformed back into native subject space using the inverse transformations of the image registration from step 1. The resulting cerebellar segmentation has a total of 28 distinct regions, outlined in Supplemental Table 1. Its performance as quantified in DICE scores are presented in Supplemental Figure 1.

| 0. Background |
| --- |
| 1. White matter |
| 2/3. Left/Right Lobules I-III |
| 4/5. Left/Right Lobule IV |
| 6/7. Left/Right Lobule V |
| 8/9. Left/Right Lobule VI |
| 10/11. Left/Right Crus I |
| 12/13. Left/Right Crus II |
| 14/15. Left/Right Lobule VIIB |
| 16/17. Left/Right Lobule VIIIA |
| 18/19. Left/Right Lobule VIIIB |
| 20/21. Left/Right Lobule IX |
| 22/23. Left/Right Lobule X |
| 24. Vermis VI |
| 25. Vermis VII |
| 26. Vermis VIII |
| 27. Vermis IX |
| 28. Vermis X |

Supplemental Table 1. Final segmentation classes.

### Training Data

The method was developed and trained on the dataset presented in Carass et al. (2018) and Diedrichsen et al. (2009) that comprised a total of 34 labeled cerebella from standard-resolution (1 mm isotropic voxel size) MRI data. All images and labels were first converted to LIA coordinates. The cerebellar labels were then cleaned by keeping only the largest connected region of each cerebellar lobule, replacing the voxels that were not connected to the largest region with the mode of the neighboring voxels. In the data set by Carass et al. (2018), lobules I-III were defined as one region and lobule IV as a separate region while lobule IV was grouped together with lobules I-III in Diedrichsen et al. (2009). To make the two datasets compatible for model training, lobule IV was grouped together with lobules I-III in the data set from Carass et al. (2018). The labels and images were then split into left and right cerebellum. The vermis was split into left and right by recursively assigning each voxel in the vermis to the mode of the neighboring voxels that had been assigned to right or left cerebellum. The images and labels were then transformed into a common space by applying the transformations resulting from registering the images to the MNI Average Brain (Collins et al., 1994). All model training took place in the common space.

### Model Training

Four different U-net models (Ronneberger et al., 2015) were used for the semantic image segmentation; one model for cerebellum masking, two for segmenting the left and right cerebellum, respectively, and one for dividing lobules I-IV into lobules I-III and lobule IV (see Figure 6). The U-net models were trained and implemented using the nnU-Net software package (Isensee et al., 2021). All models were trained using the labeled cerebella in the training data set except for the fourth model that separates lobules I-IV into lobules I-III and IV, since label IV was only labeled separately from lobules I-III marked in Carass et al. (2018), and therefore only contained a subset of 15 training samples.

## ARCUS Reconstruction

An overview of the reconstruction method is presented in Figure 7. The code for implementing this technique was done in Python 3.8, relying on the software packages Nibabel version 3.2.1 for handling neuroimaging data, Evaler for handling geometric data and ANTs version 0.2.7 for performing the non-linear registration (Avants et al., 2011; Brett et al., 2020; Samuelsson et al., 2020a). The workflow of the reconstruction (Figure 7) is described in the following steps:

1. The subject cerebellum is segmented as described above and illustrated in Figure 6.
2. The volumetric atlas is spatially resampled to the size of the subject cerebellum and registered to the subject segmentation. This is done by first assigning unique values to each region in the segmentation and correspondingly to the regions in the volumetric atlas. These two segmented volumes are then registered by the non-linear deformation procedure symmetric normalization with cross-correlation as the optimization metric as implemented in the ANTs software package (Avants et al., 2011). The transformation is saved and then applied to a virtual MRI image of the cerebellar atlas that has been created by assigning virtual contrast values to voxels according to the extent to which they lie within the cerebellar surface.
3. The image of the masked cerebellum of the subject is then normalized and the registered virtual atlas volume is registered a second time to the subject cerebellum using the same non-linear deformation technique as in step 2, but this time by contrast instead of segmentation region. The transformation resulting from this second registration is saved.
4. The first and second registration transformations from steps 2 and 3 are applied to the vertices in the surface atlas which yields the adapted cortical sheet in native subject space. Vertex normals in the surface mesh are recalculated by an unweighted average of associated face normals. Its performance, quantified in Hausdorff distance, is presented in Supplemental Figure 2.

The computational time cost of the whole segmentation and reconstruction procedures from start to finish varies with the resolution of the subject image. For the standard 1 mm^3^ resolution, i.e., the initial whole-brain input image is a 256x256x256 array, the algorithm takes about one hour using a single desktop process on an Intel Xeon Gold 6130 Processor with a base frequency of 2.10 GHz. The memory storage of the pre-processed template data required for the reconstruction is 2 GB.


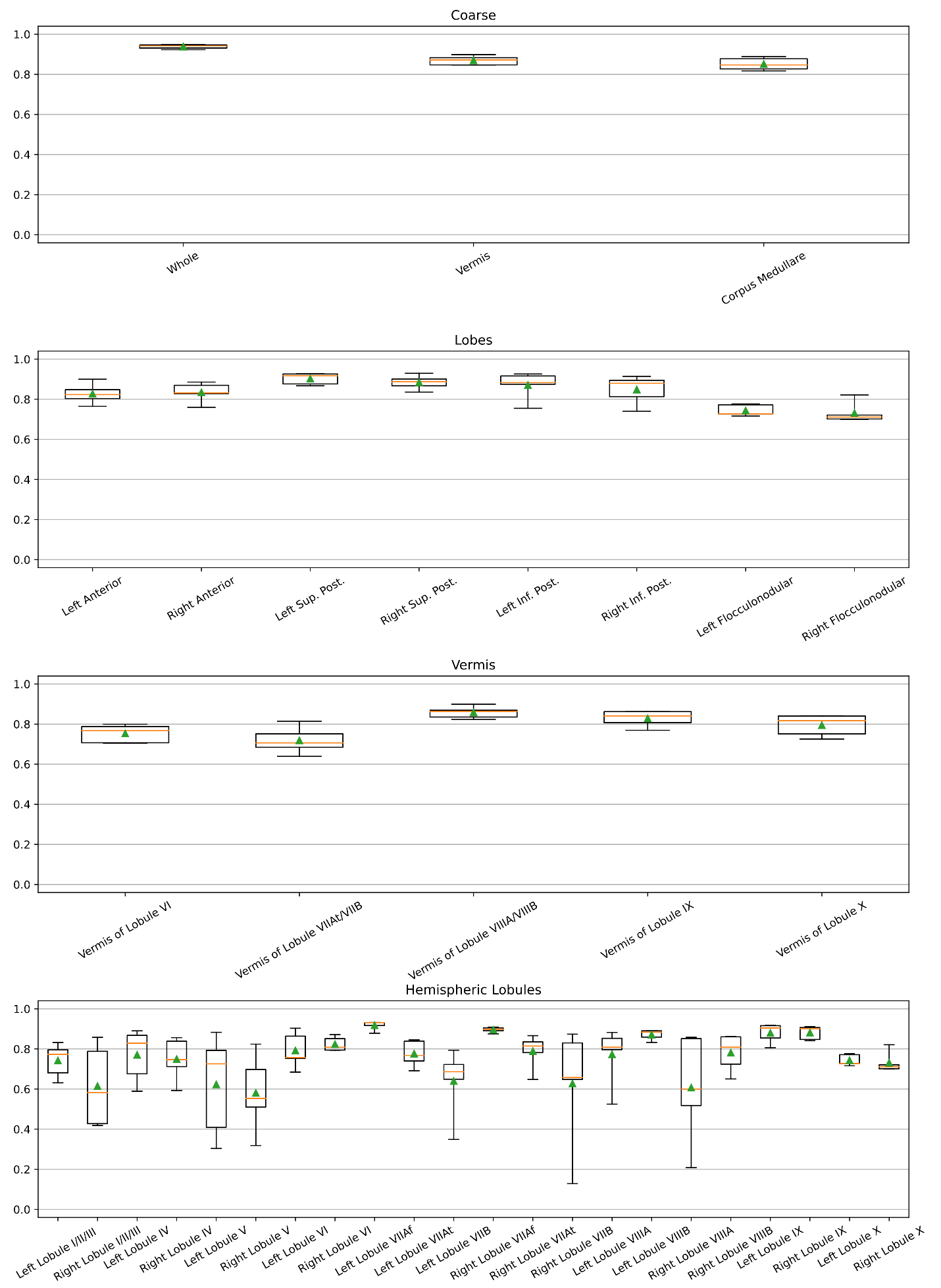


Supplemental Figure 1. Dice score distributions across subjects in three different segmentation hierarchies between predicted labels and test data. The whiskers mark the range, the boxes the interquartile range, the orange line the median and the green triangle the mean across subjects.


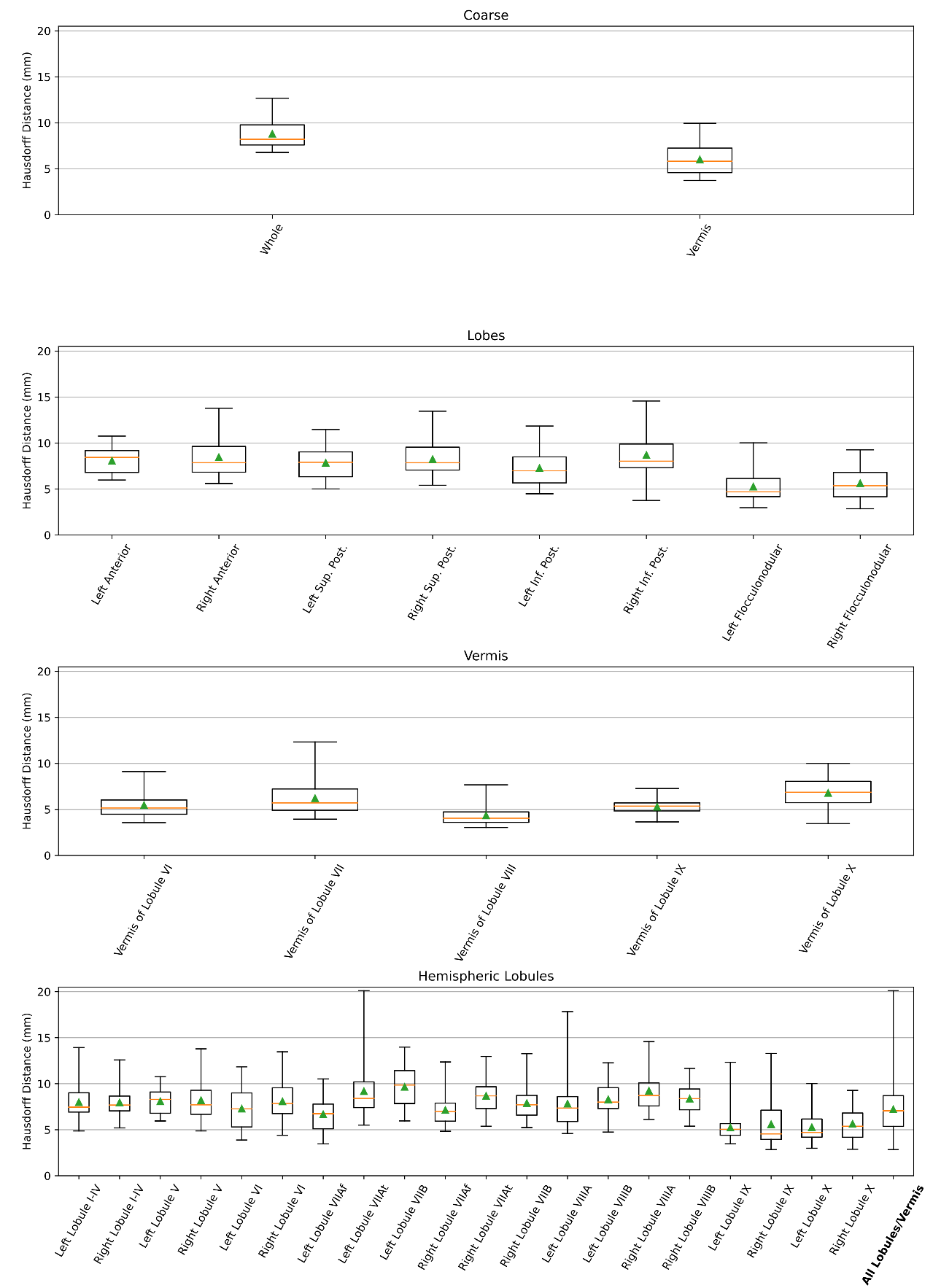


Supplemental Figure 2. Hausdorff distance distributions across subjects in three different segmentation hierarchies between the surface reconstructions and the segmentation label data. The whiskers mark the range, the boxes the interquartile range, the orange line the median and the green triangle the mean across subjects. The boxplot furthest to the right in the bottom figure includes all lobules and vermis in the two lower figures pooled together.
